# Supplementary material for: Inhibitory Effect against Listeria monocytogenes of Carbon Nanoparticles Loaded with Copper as Precursors of Food Active Packaging
Source: Foods. 2022 Sep 20;11(19):2941. doi: 10.3390/foods11192941 (PMC9562255; doi:10.3390/foods11192941)
Supplement: Supplementary file 1 [file foods-11-02941-s001.zip › Figure S2.pdf]

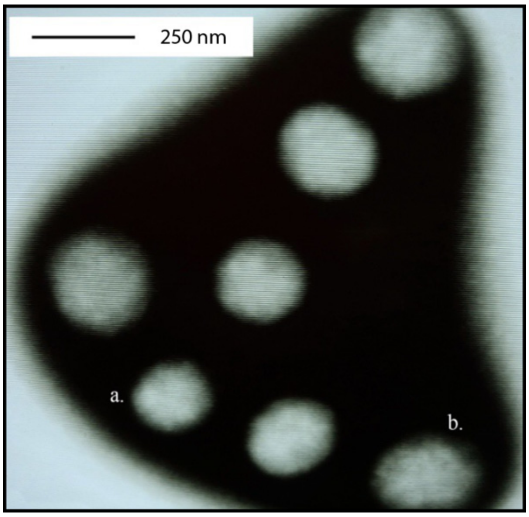

Figure S2. TEM image of LCNPs where the dimensional range of large nanoparticles is around 230 nm (letter a.), for the smallest taken into account, and 380 nm for the biggest (letter b.).
