# Supplementary figures and images for: Inhibitory Effect against Listeria monocytogenes of Carbon Nanoparticles Loaded with Copper as Precursors of Food Active Packaging
Source: Foods. 2022 Sep 20;11(19):2941. doi: 10.3390/foods11192941 (PMC9562255; doi:10.3390/foods11192941)

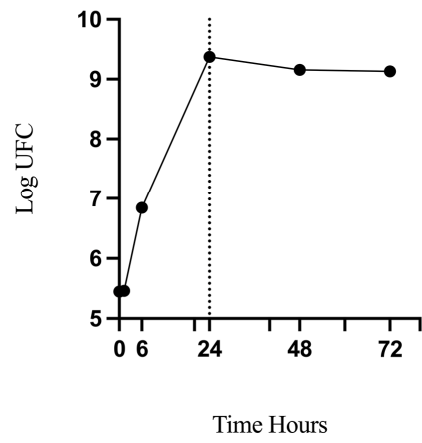

**Figure S1.** *Listeria monocytogenes* growth in optimal conditions

Supplement: Supplementary file 1 [file foods-11-02941-s001.zip › Figure S1.pdf]

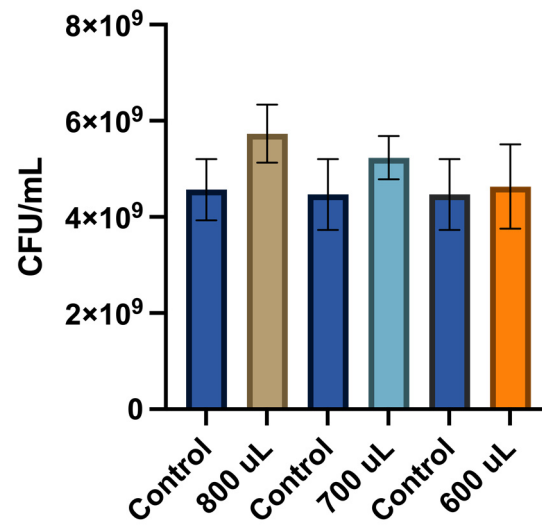

**Figure S3.** *Listeria monocytogenes*' growth in reduction of nutrients conditions

Supplement: Supplementary file 1 [file foods-11-02941-s001.zip › Figure S3.pdf]

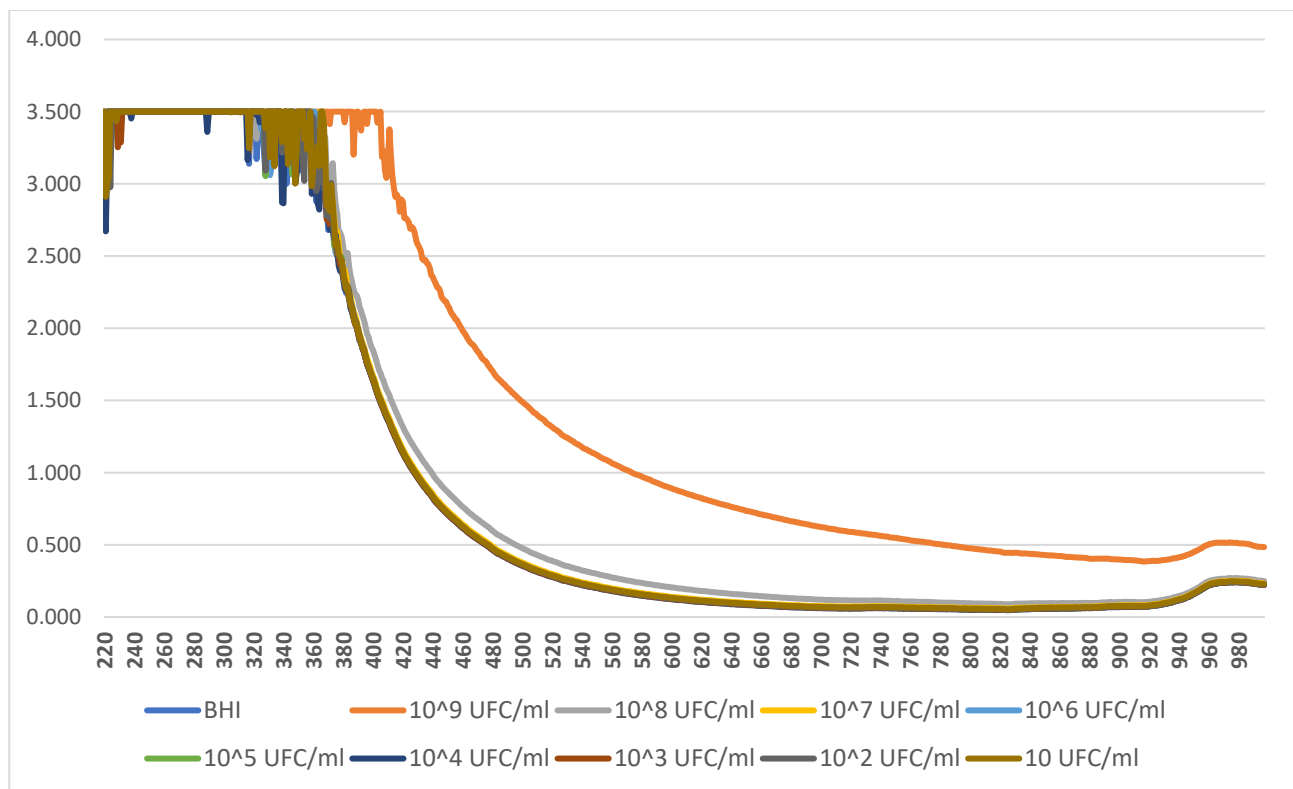

**Figure S4.** Absorbance spectra of *Listeria monocytogenes* serial dilutions

Supplement: Supplementary file 1 [file foods-11-02941-s001.zip › Figure S4.pdf]

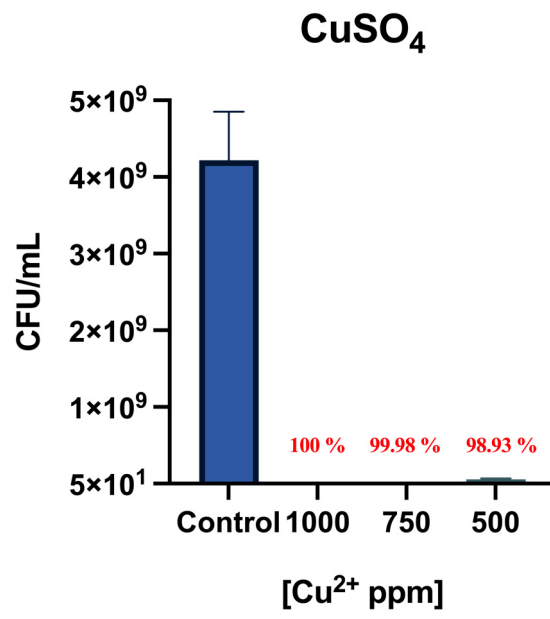

**Figure S5.** Soluble copper antimicrobial activity on *Listeria monocytogenes*

Supplement: Supplementary file 1 [file foods-11-02941-s001.zip › Figure S5.pdf]
